# Suppressive effect of AMP-activated protein kinase on the epithelial-mesenchymal transition in retinal pigment epithelial cells

**DOI:** 10.1371/journal.pone.0181481

**Published:** 2017-07-18

**Authors:** Ryo Matoba, Yuki Morizane, Yusuke Shiode, Masayuki Hirano, Shinichiro Doi, Shinji Toshima, Ryoichi Araki, Mika Hosogi, Tomoko Yonezawa, Fumio Shiraga

**Affiliations:** 1 Department of Ophthalmology, Okayama University Graduate School of Medicine, Dentistry and Pharmaceutical Sciences, Okayama, Japan; 2 Department of Molecular Biology and Biochemistry, Okayama University Graduate School of Medicine, Dentistry and Pharmaceutical Sciences, Okayama, Japan; Massachusetts Eye & Ear Infirmary, Harvard Medical School, UNITED STATES

## Abstract

The epithelial-mesenchymal transition (EMT) in retinal pigment epithelial (RPE) cells plays a central role in the development of proliferative vitreoretinopathy (PVR). The purpose of this study was to investigate the effect of AMP-activated protein kinase (AMPK), a key regulator of energy homeostasis, on the EMT in RPE cells. In this study, EMT-associated formation of cellular aggregates was induced by co-stimulation of cultured ARPE-19 cells with tumor necrosis factor (TNF)-α (10 ng/ml) and transforming growth factor (TGF)-β_2_ (5 ng/ml). 5-Aminoimidazole-4-carboxamide-1-β-D-ribofuranoside (AICAR), a potent activator of AMPK, significantly suppressed TNF-α and TGF-β_2_-induced cellular aggregate formation (p < 0.01). Dipyridamole almost completely reversed the suppressive effect of AICAR, whereas 5’-amino-5’-deoxyadenosine restored aggregate formation by approximately 50%. AICAR suppressed the downregulation of E-cadherin and the upregulation of fibronectin and α-smooth muscle actin by TNF-α and TGF-β_2_. The levels of matrix metalloproteinase (MMP)-2, MMP-9, interleukin-6, and vascular endothelial growth factor were significantly decreased by AICAR. Activation of the mitogen-activated protein kinase and mammalian target of rapamycin pathways, but not the Smad pathway, was inhibited by AICAR. These findings indicate that AICAR suppresses the EMT in RPE cells at least partially via activation of AMPK. AMPK is a potential target molecule for the prevention and treatment of PVR, so AICAR may be a promising candidate for PVR therapy.

## Introduction

Proliferative vitreoretinopathy (PVR) is one of the severe complications that can arise after rhegmatogenous retinal detachment surgery or ocular trauma. PVR is characterized by the formation of contractile fibrous membranes that cause severe tractional retinal detachment and make it difficult to reattach the retina [1]. Retinal pigment epithelial (RPE) cells are a major component of the proliferative membrane [2] and play a central role in the pathogenesis of PVR since migration and aberrant proliferation of these cells are essential for its development [3].

The epithelial-mesenchymal transition (EMT) is a process through which epithelial cells acquire a mesenchymal phenotype, and is associated with various physiological processes such as embryogenesis, as well as with pathological conditions such as tumor metastasis and fibrosis of various organs [4–6]. The EMT also plays a central role in the development of PVR, during which RPE cells undergo the EMT and transdifferentiate into myofibroblasts expressing α-smooth muscle actin (α-SMA) that produces a contractile force [7–10]. Therefore, suppressing EMT of RPE cells is considered to be a potential treatment strategy for PVR.

AMP-activated protein kinase (AMPK) consists of a catalytic subunit (α) and two regulatory subunits (β and γ), and it is a major energy sensor in eukaryotic cells. Binding of AMP to the Bateman domains on the γ subunit promotes phosphorylation at a threonine residue (Thr172) on the α subunit, inhibits dephosphorylation by protein phosphatases, and causes allosteric activation of the phosphorylated kinase. Through these three mechanisms, an increase of the AMP concentration activates AMPK, resulting in enhancement of energy production via glucose and lipid metabolism, while inhibiting anabolic processes [11–13]. In addition to its energy-sensing function, there is emerging evidence that AMPK suppresses the EMT in various types of cells, such as tubular epithelial cells [14,15], breast cancer cells [16], lung adenocarcinoma cells [17], and bronchial epithelial cells [18]. However, little has been reported regarding the effect of AMPK on RPE cells. Therefore, we performed the present study to investigate the effect of AMPK on the EMT and associated changes in RPE cells.

## Materials and methods

### Experimental reagents

Anti-fibronectin antibody and horseradish peroxidase-conjugated rabbit anti-goat IgG antibody were purchased from Abcam (Cambridge, MA, USA), FITC-conjugated anti-α-SMA antibody was purchased from Sigma (St. Louis, MO, USA), and all other antibodies were obtained from Cell Signaling (Beverly, MA, USA). Recombinant human tumor necrosis factor (TNF)-α and transforming growth factor (TGF)-β_2_ were purchased from PeproTech (Rocky Hill, NJ, USA) and R&D Systems (Minneapolis, MN, USA), respectively. 5-Aminoimidazole-4-carboxamide-1-β-D-ribofuranoside (AICAR) was obtained from Toronto Research Chemicals (North York, ON Canada). Dipyridamole (DPY) and 5’-amino-5’-deoxyadenosine (AMDA) were obtained from Sigma (St. Louis, MO, USA).

### Cell culture and EMT-associated cellular aggregate formation

A human retinal pigment epithelial cell line (ARPE-19) was obtained from the American Type Culture Collection (Manassas, VA, USA) and maintained in Dulbecco’s modified Eagle’s medium-nutrient mixture F-12 HAM (Sigma) containing 10% fetal bovine serum and 1% penicillin and streptomycin (Life Technologies, Grand Island, NY, USA) under a humidified atmosphere containing 5% CO_2_ at 37°C. The cells were plated on 35 mm dishes at a density of 3 × 10^4^ cells/cm^2^, grown to preconfluence, and starved of serum for 24 h before experiments were performed.

It has been reported that ARPE-19 cells became spindle-shaped and gathered together to form piled up cellular aggregates after culture for 48–72 h in the presence of both TNF-α and TGF-β_2_, with these aggregates being termed EMT-associated fibrotic deposits [19]. We induced these cellular aggregates according to the method described by the authors, and utilized this phenomenon as an in vitro model of the EMT.

### Enzyme-linked immunosorbent assay (ELISA)

The levels of matrix metalloproteinase (MMP)-2, MMP-9, interleukin (IL)-6, and vascular endothelial growth factor (VEGF) in culture medium were determined by using quantitative ELISA kits (R&D Systems) according to the manufacturer’s instructions. Assay values were normalized for the protein level in cell lysates.

### Western blot analysis

Western blot analysis was performed according to method described previously [20] using the following antibodies: anti-acetyl CoA carboxylase (ACC) (1:1000), anti-phospho-ACC (1:1000), anti-glyceraldehyde 3-phosphate dehydrogenase (GAPDH) (1:1000), anti-E-cadherin (1:1000), anti-fibronectin (1:5000), anti-extracellular signal-related kinase (ERK)1/2 (1:1000), anti-phospho-ERK1/2 (1:2000), anti-p38 MAPK (1:1000), anti-phospho-p38 MAPK (1:1000), anti-stress-activated protein kinase/c-Jun N-terminal kinase (SAPK/JNK) (1:1000), anti-phospho-SAPK/JNK (1:1000), anti-Smad2 (1:1000), anti-phospho-Smad2 (1:1000), anti-Smad3 (1:1000), anti-phospho-Smad3 (1:1000), anti-mTOR (1:1000), anti-phospho-mTOR (1:1000), anti-Raptor (1:1000), anti-phospho-Raptor (1:1000), anti-Tuberin/tuberous sclerosis complex 2 (TSC2) (1:1000), anti-phospho-Tuberin/TSC2 (1:1000), anti-phospho-p70 S6 kinase (p70S6K) (Thr389) (1:1000), and anti-phospho-4E-BP1 (Ser65) (1:1000). Bands were analyzed by using ImageJ software (National Institutes of Health, Bethesda, MD, USA).

### Immunocytochemistry

ARPE-19 cells were seeded and cultured on 8-well chamber slides (Thermo Fisher Scientific, Rochester, NY, USA). The cells were fixed with 4% paraformaldehyde for 10 minutes, permeabilized with 0.1% Triton X-100 for 10 minutes, blocked with 1% bovine serum albumin (Sigma) for 1 h at room temperature, and then incubated with FITC-conjugated anti-α-SMA antibody (1:100) overnight at 4°C. After being washed with phosphate-buffered saline, the nuclei were counterstained with 4’,6-diamidino-2-phenylindole (DAPI) (Life Technologies). Then the cells were mounted with Fluorescent Mounting Medium (Dako, Carpinteria, CA, USA) and examined using a Zeiss LSM 780 confocal laser scanning microscope (Carl Zeiss, Germany).

### Statistical analysis

All experiments were performed in triplicate unless otherwise noted. The t-test was used for two-group comparisons, while one-way analysis of variance (ANOVA) was employed for multiple samples. In all analyses, P < 0.05 was considered to indicate statistical significance.

## Results

### TNF-α and TGF-β_2_ cooperatively induce aggregate formation by cultured ARPE-19 cells

**Fig 1:**
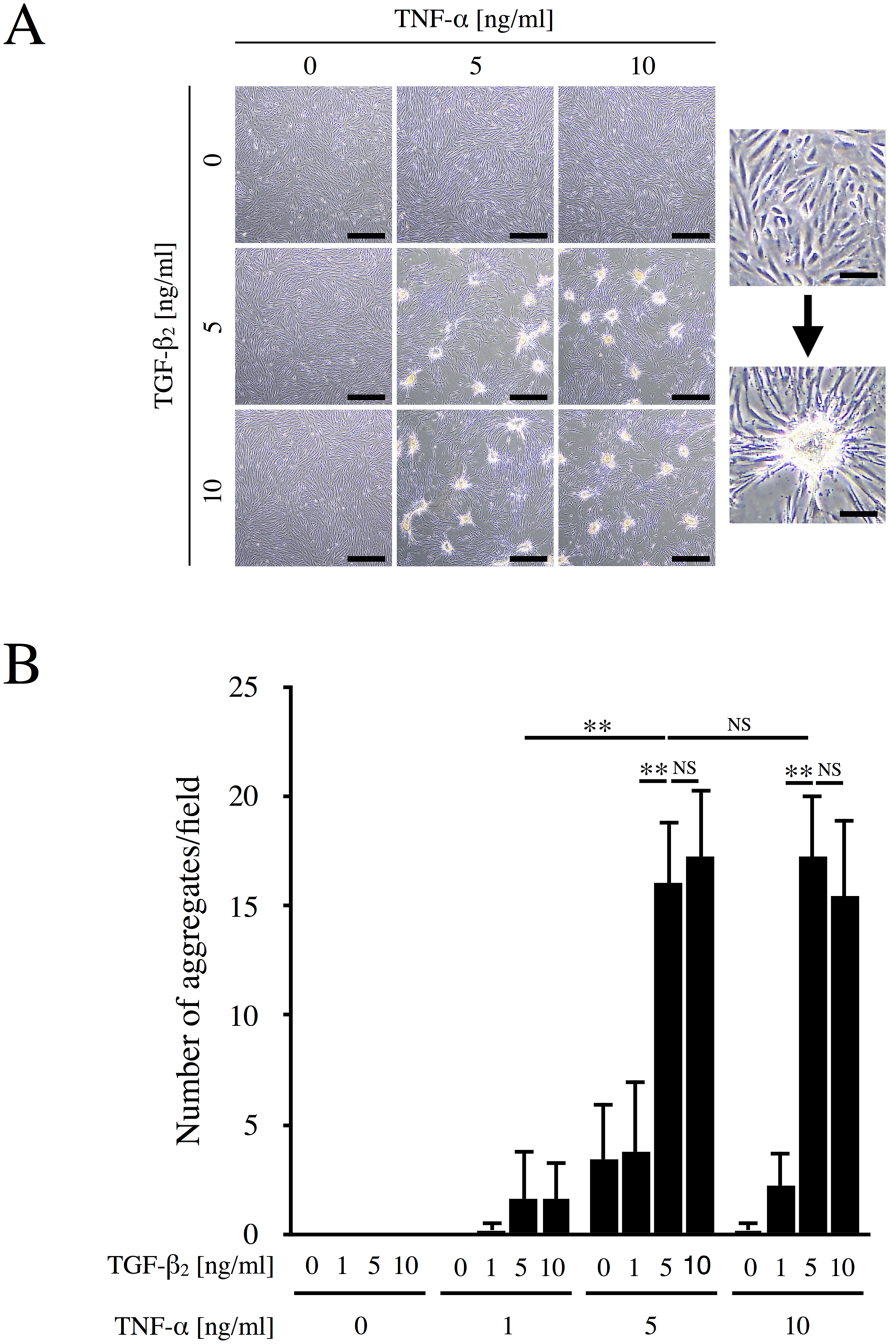
TNF-α/TGF-β_2_-induced aggregate formation by ARPE-19 cells. (A) ARPE-19 cells were cultured in the absence or presence of the indicated concentrations of TNF-α and TGF-β_2_ for 48 h. Left panel, Representative photos. Right panel, Magnified images of the cells growing as a flat monolayer or piled up in aggregates. Scale bars = 200 μm (left panel) and 40 μm (right panel). (B) The number of cellular aggregates per microscopic field was counted and analyzed (n = 5). **, p < 0.01; NS, not significant. Error bars, S.E.

It has been reported that stimulation of ARPE-19 cells with both TNF-α and TGF-β_2_ leads to formation of cellular aggregates that have been termed EMT-associated fibrotic deposits [19]. To confirm the conditions necessary for aggregate formation, we first stimulated ARPE-19 cells with various concentrations of TNF-α (0, 1, 5, and 10 ng/ml) and TGF-β_2_ (0, 1, 5, and 10 ng/ml). As shown in Fig 1A, few aggregates were observed when cells were stimulated by either TNF-α or TGF-β_2_ alone. However, the combination of TNF-α and TGF-β_2_, especially at concentrations of 5 ng/ml or more, strongly induced the formation of aggregates. Microscopic observation revealed that cultured ARPE-19 cells originally grew as a flat monolayer, but gathered together to form contractile foci after stimulation. In addition, F-actin cytoskeleton was reorganized and actin stress fibers were formed (S1 Fig). Fig 1B shows the number of cellular aggregates per microscopic field. No aggregates were observed when RPE cells were cultured in the presence of TGF-β_2_ alone. On the other hand, 5 ng/ml TNF-α alone induced a few aggregates, while aggregates were markedly enhanced when the cells were concomitantly incubated with 5 ng/ml TGF-β_2_ (p < 0.01). Based on these findings and the previous report [19], subsequent experiments were conducted using 10 ng/ml TNF-α plus 5 ng/ml TGF-β_2_ (TNF-α/TGF-β_2_) to stimulate RPE cells.

### AICAR suppresses TNF-α/TGF-β_2_-induced aggregate formation

**Fig 2:**
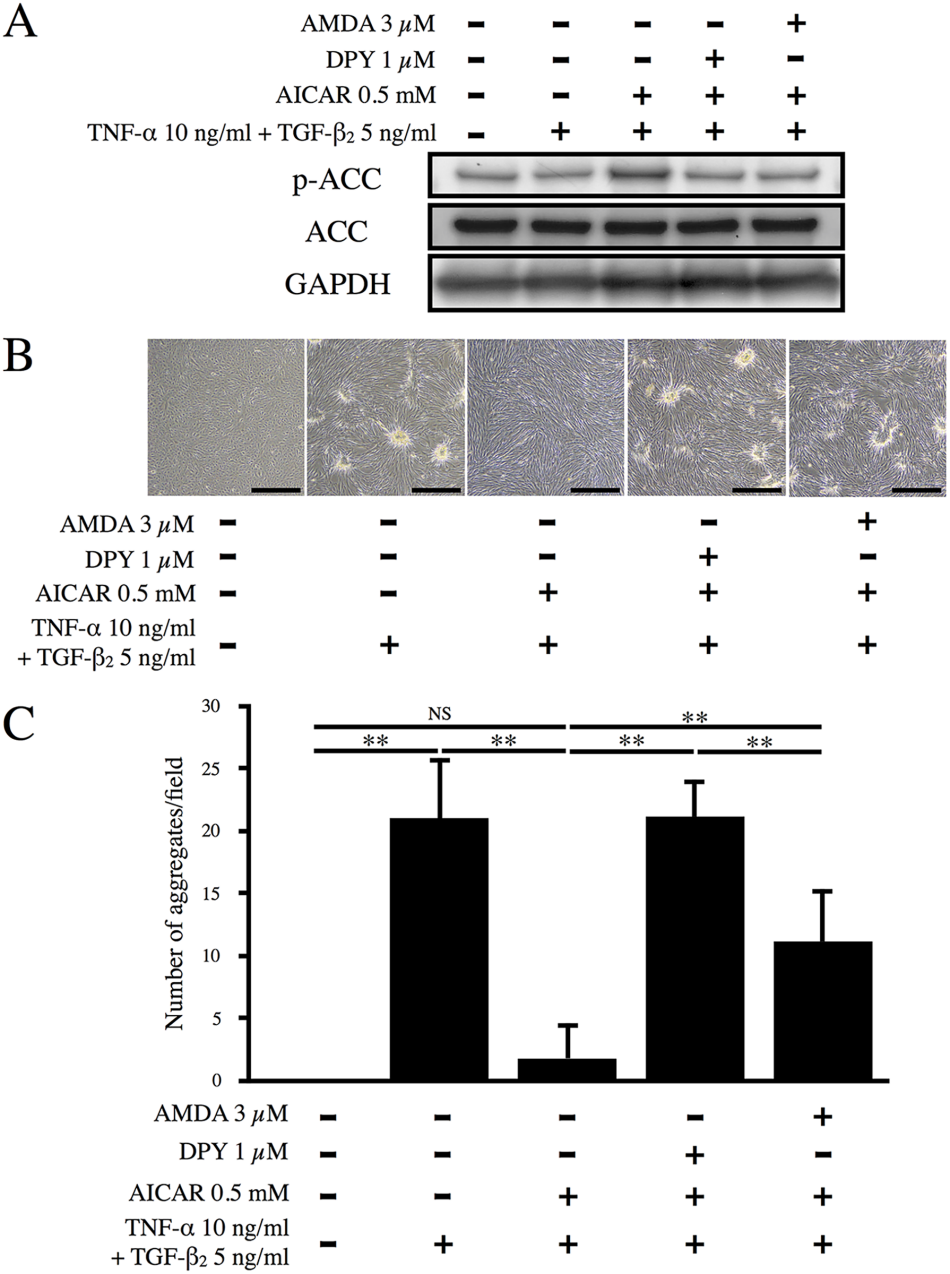
Effect of AICAR on TNF-α/TGF-β_2_-induced aggregate formation. (A) Phosphorylation of ACC in whole cell lysates was examined by western blot analysis. GAPDH was used as a loading control. (B) ARPE-19 cells were cultured with or without TNF-α and TGF-β_2_, AICAR, DPY, or AMDA for 48 h. Representative photos are shown. Scale bars = 500 μm. (C) The number of aggregates per microscopic field was counted and analyzed (n = 6). **, p < 0.01; NS, not significant. Error bars, S.E.

We investigated the effect of AICAR (an AMPK activator) on formation of aggregates by ARPE-19 cells, as well as the effects of two AICAR inhibitors (DPY and AMDA). The effect of these reagents on AMPK was evaluated by monitoring the phosphorylation of ACC, a well-known substrate of AMPK. As shown in Fig 2A, co-stimulation with TNF-α/TGF-β_2_ did not affect the phosphorylation of ACC. AICAR increased the phosphorylation of ACC, while DPY and AMDA inhibited it, indicating that these reagents regulated the activation of AMPK. As shown in Fig 2B and 2C, incubation of RPE cells with TNF-α/TGF-β_2_ significantly induced the formation of aggregates (p < 0.01), while aggregate formation was dramatically suppressed by AICAR (p < 0.01). DPY inhibits adenosine transporters to prevent AICAR from entering cells. Incubation of RPE cells with DPY almost completely restored aggregate formation after it had been suppressed by AICAR (p < 0.01), indicating that this effect of AICAR is mediated by intracellular pathways. AMDA blocks the conversion of AICAR to ZMP (a monophosphorylated form that activates AMPK by mimicking AMP) by inhibiting adenosine kinases, and it also significantly inhibited the suppressive effect of AICAR, although its effect was approximately half that of DPY. These results suggest that AICAR suppressed aggregate formation by ARPE-19 cells at least partly via the activation of AMPK. Furthermore, we also studied the effect of DMEM/F-12 medium, which is known to promote the fibroblastic phenotype, on ARPE-19 cells. The resulting cellular aggregates in DMEM were fewer and appeared to be milder than those in DMEM/F-12. Moreover, it was evident that TNF-α/TGF-β_2_ significantly elevated the number of aggregates in DMEM and DMEM/F-12 media, while AICAR significantly suppressed it (S2 Fig). Therefore, this suggested that the suppressive effect of AICAR on EMT was independent of culture medium.

### AICAR suppresses induction of the EMT by TNF-α/TGF-β_2_

**Fig 3:**
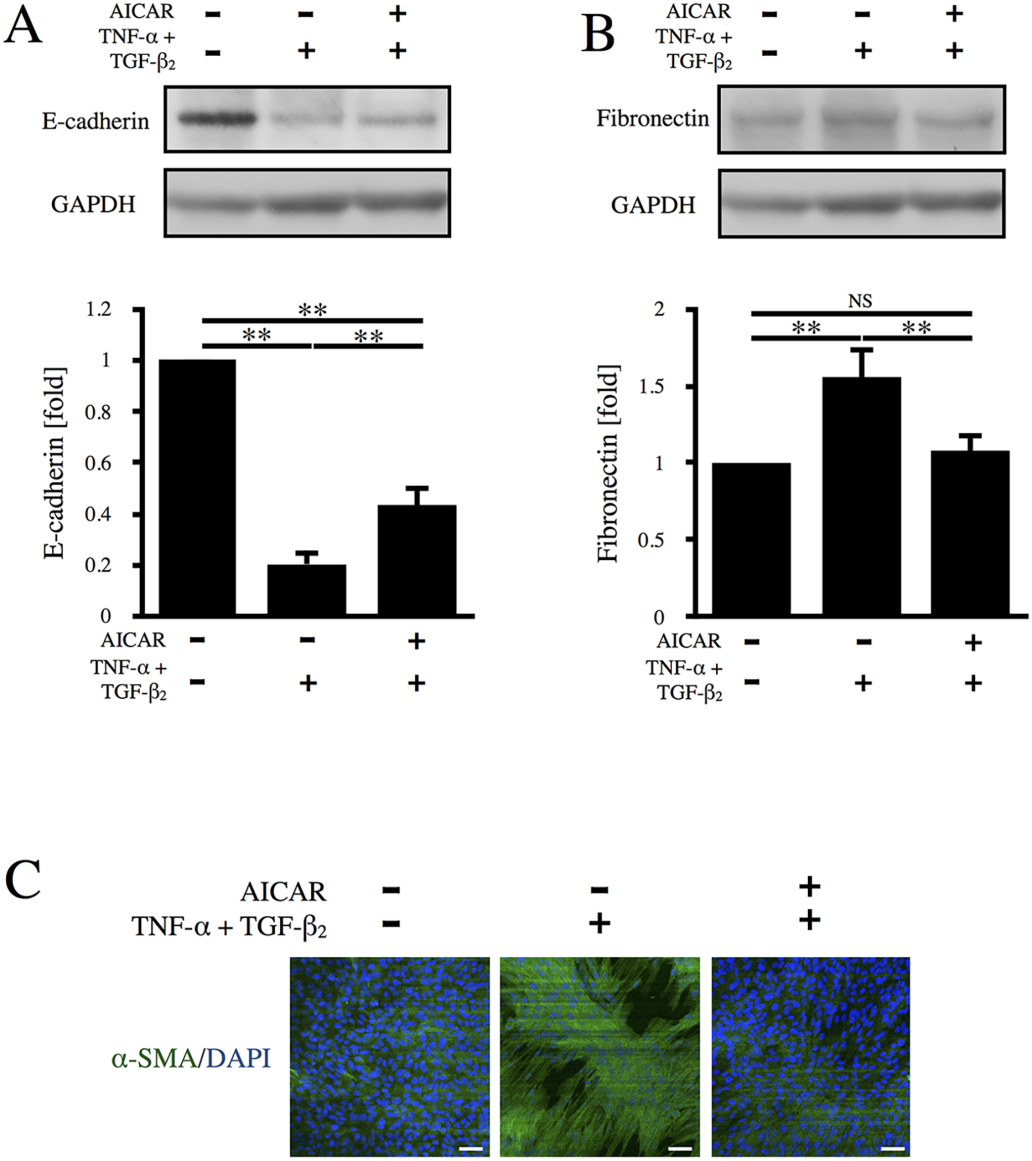
Effect of AICAR on the TNF-α/TGF-β_2_-induced EMT. Levels of E-cadherin (A) and fibronectin (B) were determined by western blot analysis. (C) Representative images of immunocytochemical staining for α-SMA. Nuclei were counterstained with DAPI. Scale bars = 50 μm.

Formation of aggregates by RPE cells is considered to be an indicator of the EMT-associated fibrotic response [19]. Therefore, we focused on the effect of AICAR on the EMT and evaluated the expression of EMT-related proteins. As shown in Fig 3A, incubation of RPE cells with TNF-α/TGF-β_2_ induced the downregulation of E-cadherin (a well-known epithelial marker protein), indicating the occurrence of the EMT. Pretreatment of cells with AICAR significantly restored E-cadherin expression (p < 0.01). Similarly, the expression of RPE65 and cellular retinaldehyde-binding protein (CRALBP), RPE-specific markers, was decreased by TNF-α/TGF-β_2_, while it was restored by AICAR (S3 Fig). On the other hand, expression of fibronectin (a representative mesenchymal marker) was upregulated by TNF-α/TGF-β_2_, while it was significantly suppressed by AICAR (p < 0.01) (Fig 3B). RPE cells are known to undergo transdifferentiation into myofibroblasts via the EMT and express α-SMA [9], so we next investigated the expression of α-SMA. As shown in Fig 3C, α-SMA expression was strongly enhanced by incubation of RPE cells with TNF-α/TGF-β_2_, suggesting that the cells had undergone transdifferentiation into myofibroblasts via the EMT. Elevated expression of α-SMA was reversed by treatment of the cells with AICAR. Taken together, these results indicate that AICAR suppressed induction of the EMT in RPE cells by TNF-α/TGF-β_2_.

### AICAR suppresses TNF-α/TGF-β_2_-induced upregulation of MMP-2, MMP-9, IL-6, and VEGF

**Fig 4:**
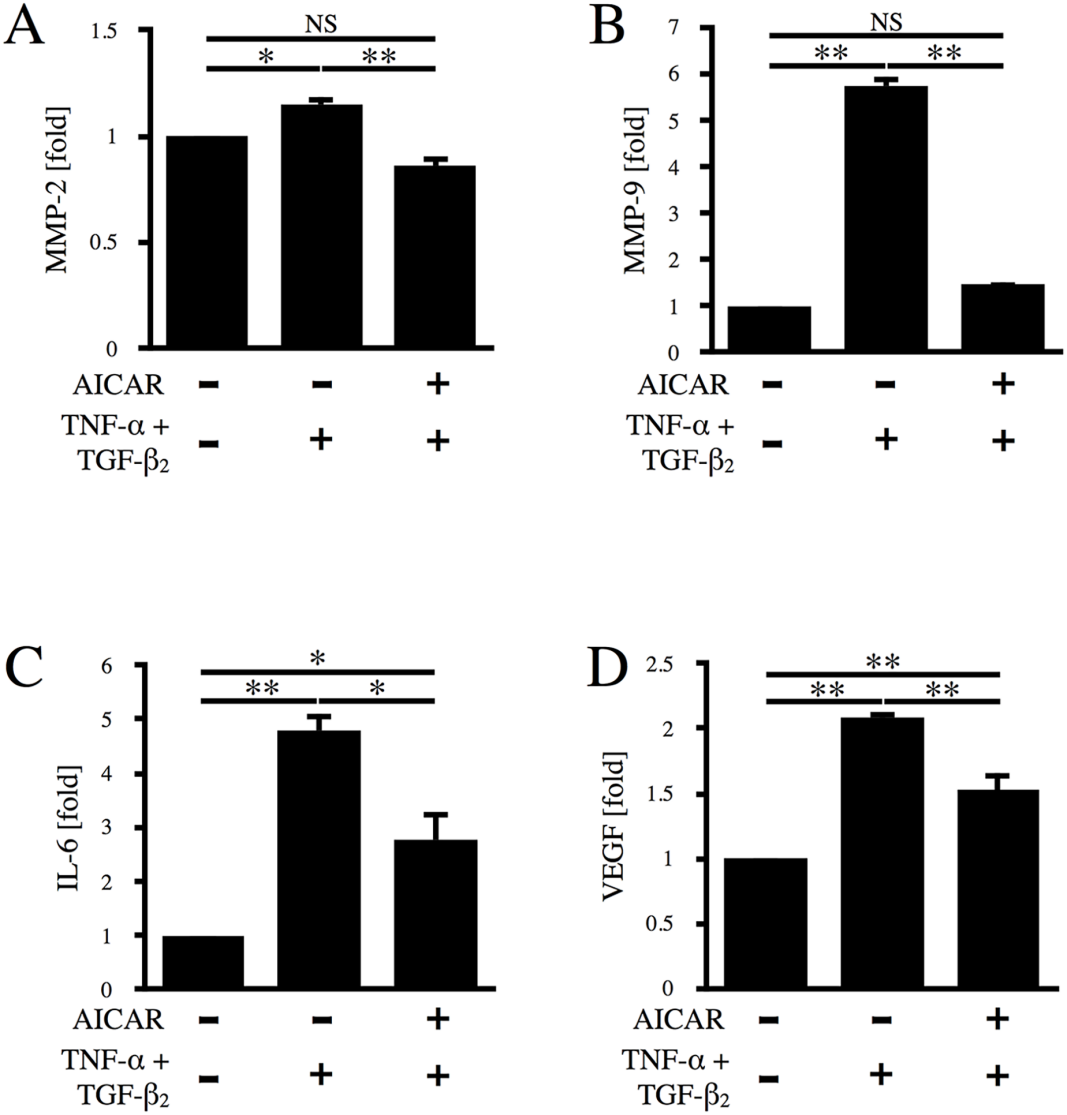
Effect of AICAR on TNF-α/TGF-β_2_-induced upregulation of MMP-2, MMP-9, IL-6, and VEGF. The levels of MMP-2 (A), MMP-9 (B), IL-6 (C), and VEGF (D) in culture medium were determined by ELISA after 24 h of stimulation with TNF-α/TGF-β_2_ in the absence or presence of AICAR. *, p < 0.05; **, p < 0.01; NS, not significant. Error bars, S.E.

Since various cytokines, proteases, and growth factors (including TNF-α, TGF-β, MMP-2, MMP-9, IL-6, and VEGF) are known to be involved in the development of PVR [21], we next evaluated the levels of MMP-2, MMP-9, IL-6, and VEGF in RPE cell culture medium by ELISA. As shown in Fig 4A and 4B, incubation of RPE cells with TNF-α/TGF-β_2_ significantly increased the levels of MMP-2 and MMP-9 in culture medium compared to the control by 1.15-fold (p < 0.05) and 5.74-fold (p < 0.01), respectively, while AICAR almost completely suppressed the elevation of MMP-2 and MMP-9 (p < 0.01). As shown in Fig 4C and 4D, incubation with TNF-α/TGF-β_2_ elevated the levels of IL-6 and VEGF by 4.79-fold (p < 0.01) and 2.09-fold (p < 0.01), respectively. Although AICAR did not completely suppress IL-6 and VEGF production, it was significantly reduced (p < 0.05 and p < 0.01, respectively).

### AICAR suppresses MAPK signaling

**Fig 5:**
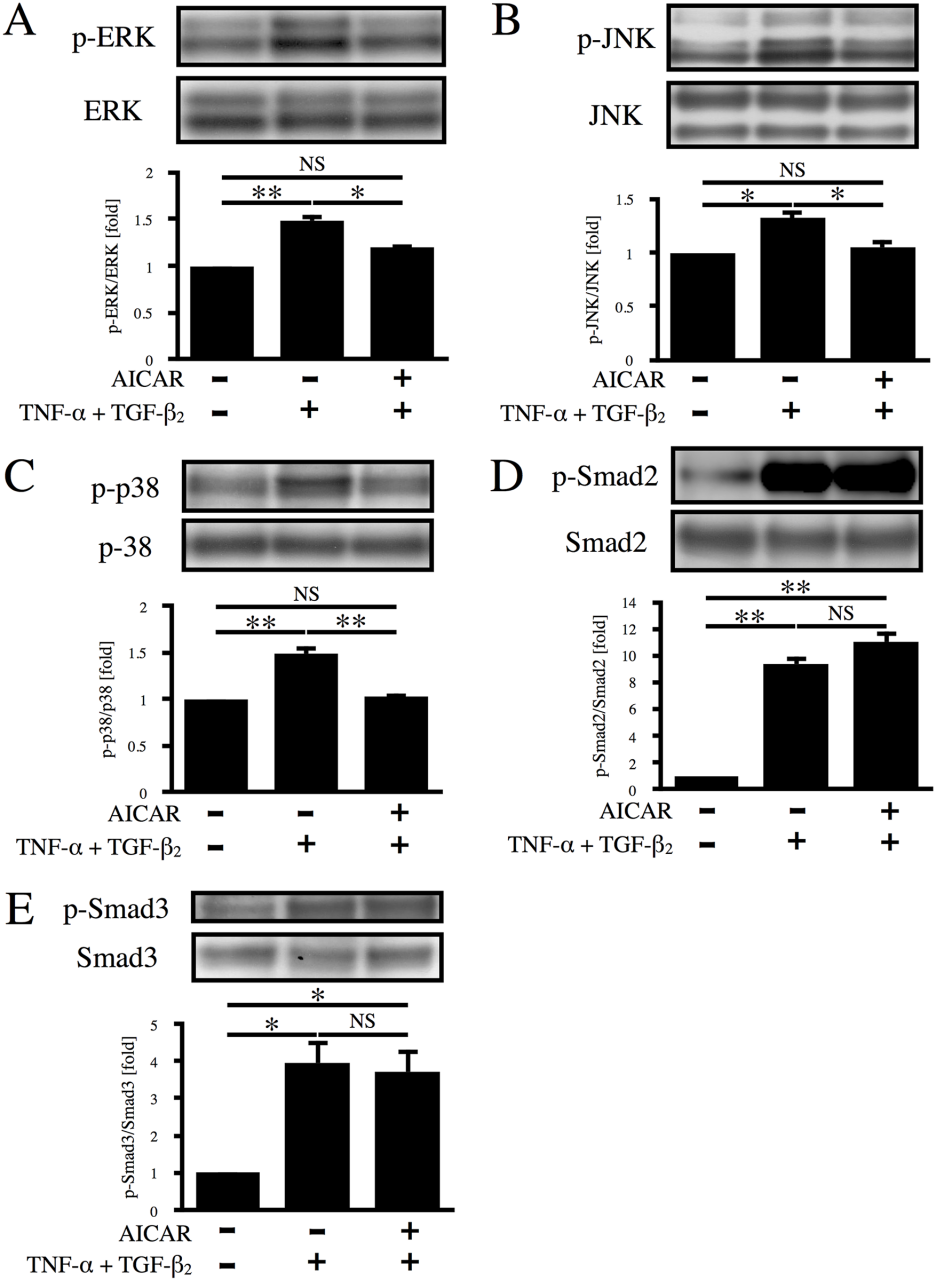
Effect of AICAR on the MAPK and Smad signaling pathways. Phosphorylation of ERK (A), JNK (B), p38 (C), Smad2 (D), and Smad3 (E) was examined by western blot analysis after 12 h of stimulation with TNF-α/TGF-β_2_ in the absence or presence of AICAR. *, p < 0.05; **, p < 0.01; NS, not significant. Error bars, S.E.

TNF-α is a proinflammatory cytokine with multiple biological functions related to inflammation, apoptosis, and cell proliferation that activates various downstream cascades, including the MAPK pathways that may be associated with proliferation and migration of RPE cells [22–27]. It has also been established that TGF-β is involved in the EMT and ocular fibrotic diseases, such as PVR and proliferative diabetic retinopathy, mainly via Smad pathways [28,29]. To elucidate the signaling pathways responsible for the suppressive effect of AICAR on aggregation of RPE cells, phosphorylation of ERK, JNK, p38, Smad2, and Smad3 was investigated by western blot analysis. As shown in Fig 5A–5C, co-stimulation with TNF-α/TGF-β_2_ increased the phosphorylation of ERK, JNK, and p38 MAPK by 1.48-, 1.33-, and 1.48-fold, respectively (p < 0.05), while phosphorylation of these MAPKs was almost completely suppressed by AICAR. Treatment of RPE cells with TNF-α/TGF-β_2_ also elevated the phosphorylation of Smad2 and Smad3 by 9.40- and 3.43-fold, respectively. In contrast to its effect on the MAPKs, AICAR did not suppress phosphorylation of either Smad2 or Smad3 (Fig 5D and 5E). Considering these results, MAPKs seem to be involved in suppression of the EMT by AICAR, while Smad2 and Smad3 have no role.

### Suppression of TNF-α/TGF-β_2_-induced aggregate formation by MAPK inhibition

**Fig 6:**
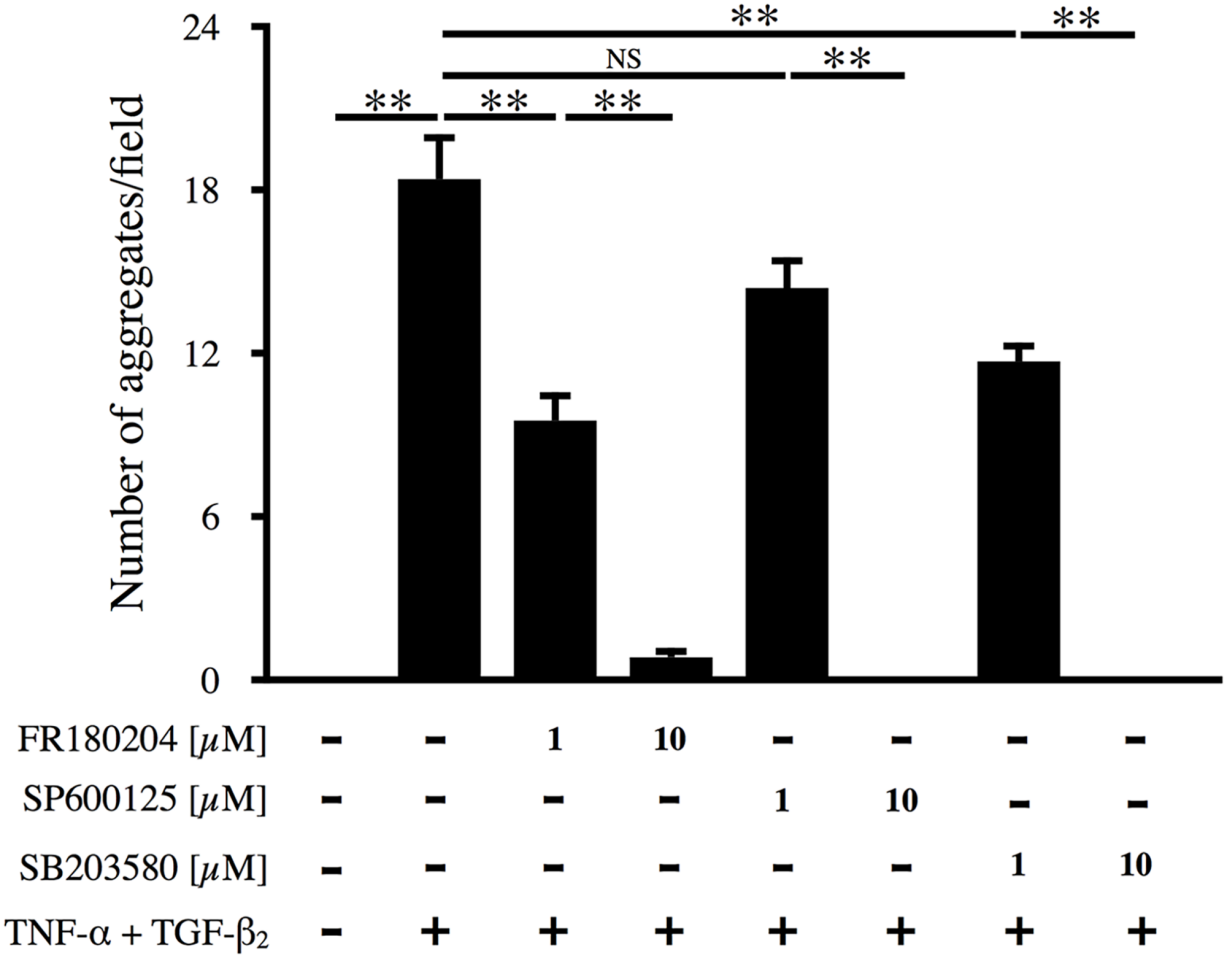
Effect of MAPK inhibitors on aggregate formation. Inhibitors of ERK (FR18004), JNK (SP600125), and p38 (SB203580) were added to the culture medium at 1 h before TNF-α/TGF-β_2_ co-stimulation. The number of cellular aggregates per microscopic field was counted and analyzed (n = 6).

To further investigate the association of MAPKs with the formation of RPE cell aggregates, inhibitors of ERK (FR180204), JNK (SP600125), and p38 (SB203580) were added to the culture medium at 1 h before stimulation of cells with TNF-α/TGF-β_2_. Each of the inhibitors significantly suppressed aggregate formation in a concentration-dependent manner (Fig 6; p < 0.01), suggesting that activation of all of these pathways (ERK, JNK, and p38) is essential for the formation of RPE cell aggregates.

### AICAR suppresses the mTOR pathway

**Fig 7:**
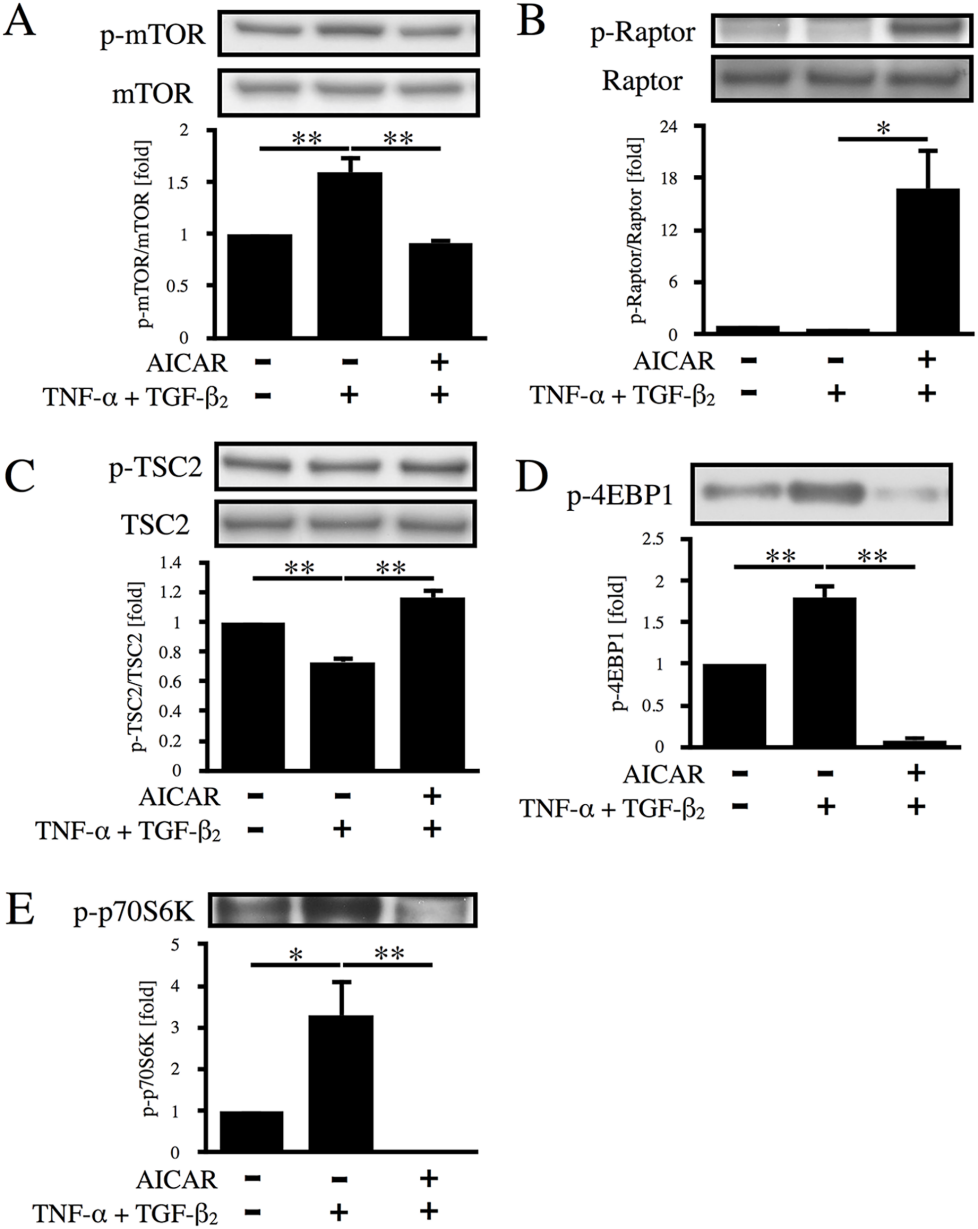
Effect of AICAR on the mTOR signaling pathway. Phosphorylation of mTOR (A), Raptor (B), TSC2 (C), 4EBP1 (D), and p70S6K (E) was examined by western blot analysis after 12 h of stimulation with TNF-α/TGF-β_2_ in the absence or presence of AICAR. *, p < 0.05; **, p < 0.01; Error bars, S.E.

Uncontrolled proliferation of RPE cells plays a central role in the pathogenesis of PVR and is also important for aggregate formation. The mTOR pathway is known to be one of the key regulators of cell proliferation [30]. Since it has been established that AMPK activation leads to inhibition of the mTOR pathway, we studied the effects of AICAR on the mTOR pathway in RPE cells. As shown in Fig 7, treatment of the cells with TNF-α/TGF-β_2_ promoted mTOR phosphorylation via dephosphorylation of Raptor and TSC2, resulting in the phosphorylation of 4E-BP1 and p70S6K. In contrast, AICAR increased the phosphorylation of Raptor and TSC2, caused inactivation of mTOR, and significantly decreased the expression of p-4E-BP1 and p-p70S6K (p < 0.01).

## Discussion

In the present study, we utilized an in vitro model that involved co-stimulation of RPE cell monolayers with TNF-α and TGF-β_2_, resulting in the formation of cellular aggregates, and we demonstrated that AICAR almost completely suppressed this phenomenon. Formation of aggregates by RPE cells occurred because of the EMT and not because of natural mechanisms occurring within epithelial cells, as demonstrated by Takahashi et al. [16] and by our present findings (Fig 3 and S3 Fig). During EMT, E-cadherin downregulation leads to a decrease in cell-cell adhesion and a loss of apical-basal polarity. Actin cytoskeleton reorganization causes cell elongation and increases cell motility. In addition, the remodeling of extracellular matrix proteins such as fibronectin is enhanced. These mechanisms increase cell migration and invasion [4]. Present findings suggested that ARPE-19 cells underwent EMT via MAPK and Smad pathways activated by TNF-α/TGF-β_2_ co-stimulation, and then migrated and gathered together to form piled up cellular aggregates through the mechanisms highlighted above. Conversely, no cellular aggregates were formed in primary RPE cells under the same condition. However, once a monolayer of primary RPE cells, which was treated with TNF-α and TGF-β_2_, was wounded by a scratch, migrated cells formed piled up cellular aggregates. As was the case with ARPE-19 cells, AICAR suppressed TNF-α/TGF-β_2_-induced cellular aggregate formation (S4 Fig). The evidence suggested that an additional condition like the disruption of cell-cell contact was required to cause EMT-associated aggregate formation by primary RPE cells. RPE cells undergo the EMT in various pathological conditions, including PVR and proliferative diabetic retinopathy, in which contractile proliferative membranes are created by the migration and uncontrolled proliferation of transformed RPE cells, leading to untreatable tractional retinal detachment. Hence, our findings suggest that AICAR could be a novel candidate treatment for EMT-related diseases.

We also demonstrated that AICAR exerts a suppressive effect on the EMT of RPE cells at least partially via activation of AMPK. The mechanisms through which AICAR acts have already been extensively investigated. After translocation into the cell via adenosine transporters, AICAR acts through both non-AMPK and AMPK pathways. The former mechanisms include inhibition of S-adenosylmethionine-dependent methylation reactions [31], interference with the binding of DNA by transcription factors such as NF-κB [32], and inhibition of the PI 3-kinase/Akt pathway [33]. The latter mechanism requires conversion of AICAR to ZMP by adenosine kinases, after which ZMP mimics AMP and stimulates the phosphorylation of AMPK. Therefore, to clarify whether or not AMPK was involved in the suppressive effect of AICAR on the EMT in RPE cells, we utilized two AICAR inhibitors (DPY and AMDA), and evaluated AMPK activation from downstream phosphorylation of ACC. When DPY was used to inhibit the entry of AICAR into the RPE cells, aggregate formation was almost completely restored, indicating that AICAR acted via intracellular pathways. In contrast, approximately 50% of aggregate formation was restored by using AMDA to block the conversion of AICAR to ZMP, suggesting that AMPK is at least partially responsible for the effects of AICAR. Moreover, we showed that the mTOR pathway, a well-known downstream target of AMPK, and its effectors (4E-BP1 and p70S6K) were significantly suppressed by AICAR (Fig 7), suggesting that regulation of cell proliferation via the AMPK-mTOR axis plays a role in the suppression of aggregate formation by AICAR. Further investigations will be needed to clarify the mechanisms underlying the partial inhibitory effect of AICAR that was not mediated by AMPK activation.

Our findings demonstrated that AICAR suppresses some of the factors associated with PVR, supporting its possible effectiveness for treating this disease. Various cytokines, growth factors, and proteases have been reported to be involved in the pathogenesis of PVR, including MMP-2, MMP-9, IL-6, and VEGF [34–40]. In particular, we found that the MMP-9 was markedly increased by TNF-α/TGF-β_2_ co-stimulation of RPE cells, while it was almost completely suppressed by AICAR (Fig 4B), suggesting that MMP-9 may have a pivotal role in the transdifferentiation of RPE cells and may be one of the primary targets for AICAR. This result is in accord with the previous report by Morizane et al. that MMP-9 expression was suppressed by activation of AMPK in mouse embryonic fibroblasts [20]. In contrast, the effect of AICAR on MMP-2 was less marked, although still statistically significant (Fig 4A), suggesting that MMP-2 plays a somewhat lesser role in RPE cell transdifferentiation compared with MMP-9. Incubation of RPE cells with TNF-α/TGF-β_2_ also led to significant elevation of IL-6 and VEGF levels. Although AICAR significantly reduced the levels of both IL-6 and VEGF, these factors were only partly suppressed, suggesting a less important role than that of MMP-9 in the suppressive effect of AICAR on EMT-based aggregate formation by RPE cells.

The present study also showed that MAPKs, but not the Smad pathway, are important for the suppressive effect of AICAR on aggregate formation by RPE cells. TNF-α/TGF-β_2_ co-stimulation of RPE cells led to phosphorylation of ERK, JNK, p38, Smad2, and Smad3, consistent with previous reports [22,41]. AICAR significantly suppressed the phosphorylation of all three MAPKs. This result is compatible with previous reports that activation of AMPK by AICAR suppresses MAPKs in various types of cells [42–44]. Also, aggregation of RPE cells was dose-dependently suppressed by pretreatment with either an ERK, JNK, or p38 inhibitor, suggesting that all of these MAPKs are important for the EMT of RPE cells. Interestingly, AICAR did not affect phosphorylation of Smad2 and Smad3, although the Smad pathway is known to play a central role in the TGF-β-mediated EMT [9]. This result may reasonably be explained by the different phosphorylation sites of Smad proteins. Smad2 and Smad3 can be activated by both TNF-β and other cytokines. TGF-β binds to its receptor and then activates Smad2 and Smad3 by phosphorylating the carboxyl-terminal region. Phosphorylated Smad2 and Smad3 subsequently form a heteromeric complex with Smad4, followed by translocation to the nucleus to regulate gene expression. Recent studies have shown that MAPKs phosphorylate Smad2 and Smad3 in the middle linker region, rather than the carboxyl-terminal region, to activate Smad signaling [28]. In the present study, we performed immunoblotting with antibodies that specifically detected Smad2 and Smad3 with carboxyl-terminal phosphorylation. Taken together, it is possible that formation of RPE cell aggregates requires full activation of the Smad pathway through phosphorylation of the middle linker regions of Smads by MAPKs, and that AICAR acts by suppressing MAPK phosphorylation and thus preventing complete Smad pathway activation. Further studies will be required to fully elucidate the signaling pathways associated with AICAR.

**Fig 8:**
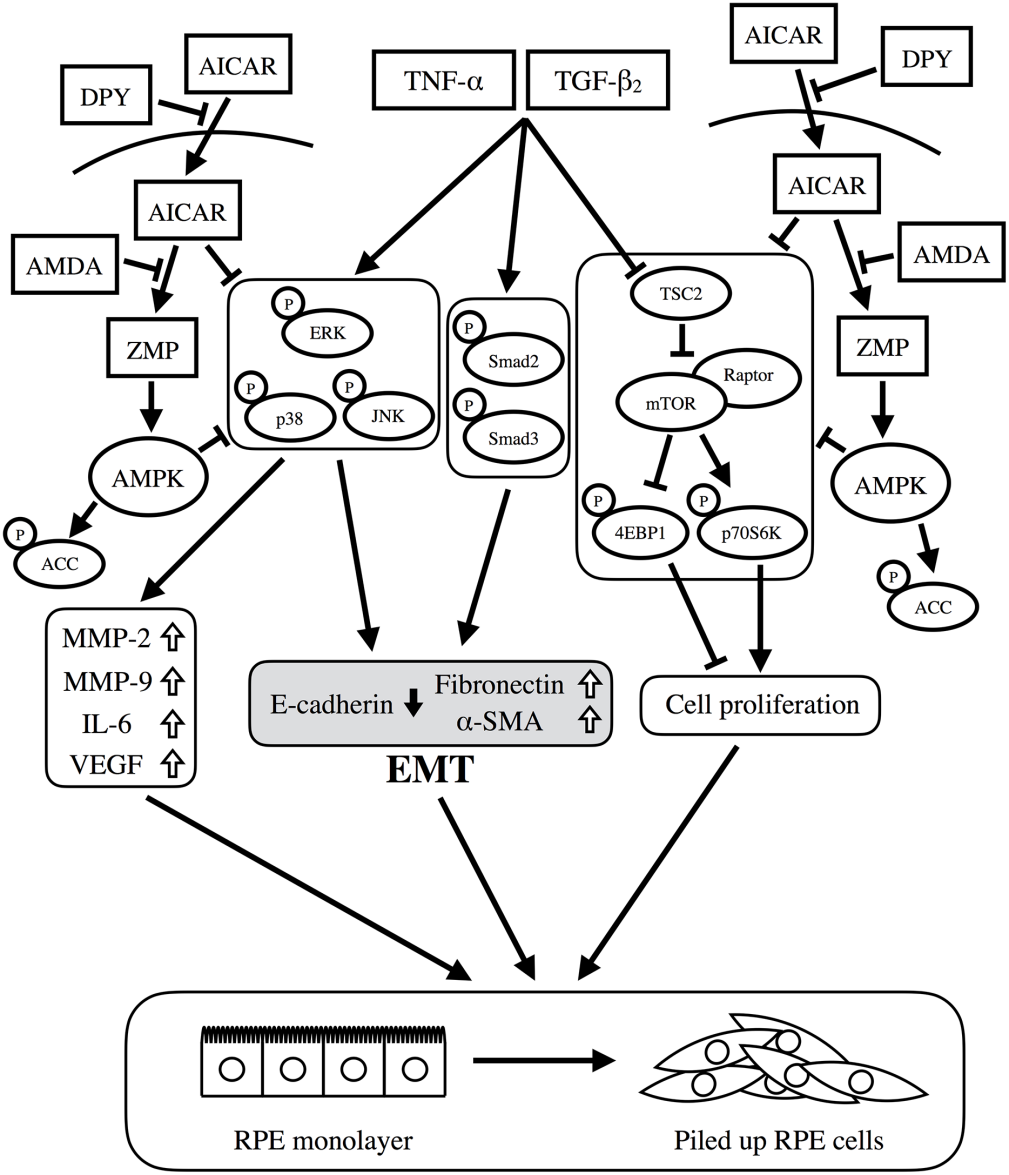
Proposed signaling pathways relevant to the effects of AICAR. TNF-α/TGF-β_2_ co-stimulation activates the ERK, JNK, and p38MAPK pathways, Smad2/3 pathway, and mTOR pathway. Activation of the MAPKs and the Smad pathway induces EMT (downregulation of E-cadherin with upregulation of fibronectin and α-SMA), and also upregulates MMPs and inflammatory cytokines such as IL-6 and VEGF. Activation of the mTOR pathway leads to cell proliferation. These changes disrupt the static RPE cell monolayer, leading to the formation of piled up cellular aggregates. When AICAR enters a cell, it is converted to ZMP, which mimics AMP that in turn activates AMPK and its downstream target, ACC. DPY prevents AICAR from entering the cell; AMDA inhibits AICAR’s conversion to ZMP. AICAR suppresses aggregate formation by inhibiting the ERK, JNK, p38 MAPK, and mTOR pathways, at least partially via activation of AMPK.

In conclusion, we demonstrated that AICAR suppressed the EMT and aggregate formation by RPE cells along with upregulation of MMP-2, MMP-9, IL-6, and VEGF at least partially via activation of AMPK (Fig 8). Thus, AMPK is a potential target molecule for the prevention and treatment of PVR, and AICAR may be a promising candidate for PVR therapy.

## Supporting information

S1 Fig Actin cytoskeleton reorganization after TNF-α/TGF-β_2_ treatment. ARPE-19 cells were cultured with or without TNF-α/TGF-β_2_ for 48 h and stained with Acti-stain^™^ 488 phalloidin (Cytoskeleton, Inc.). Nuclei were counterstained with DAPI. Representative photos are shown. Scale bars = 20 μm.
(PDF)

S2 Fig Comparison of the number of TNF-α/TGF-β_2_-induced cellular aggregates with or without F-12. (A) ARPE-19 cells were cultured in the absence or presence of TNF-α/TGF-β_2_ and AICAR for 48 h in DMEM/F-12 or DMEM. Left, Representative images. Arrowheads indicate piled up cellular aggregates. Right, Magnified images of the aggregate. Scale bars = 200 μm (left images) and 50 μm (right magnified images). (B) Comparison of the number of TNF-α/TGF-β_2_-induced cellular aggregates with or without F-12. The number of aggregates per microscopic field was counted. **, p < 0.01. Error bars, S.E. (C) Comparison of the suppressive effect of AICAR on the TNF-α/TGF-β_2_-induced cellular aggregate formation with or without F-12. The number of aggregates per microscopic field was counted and analyzed. **, p < 0.01; NS, not significant. Error bars, S.E.
(PDF)

S3 Fig Effect of AICAR on TNF-α/TGF-β_2_-induced downregulation of RPE65 and CRALBP. (A) Levels of RPE65 and CRALBP were determined by western blot analysis. GAPDH was used as a loading control. (B) ARPE-19 cells cultured with or without TNF-α/TGF-β_2_ and AICAR for 48 h were fixed and stained using antibodies against RPE65 and CRALBP (Abcam). Nuclei were counterstained with DAPI. Representative photos are shown. Scale bars = 20 μm.
(PDF)

S4 Fig Aggregate formation by primary RPE cells after TNF-α/TGF-β_2_ stimulation with scratch wound assay. Primary RPE cells were cultured to confluence. The monolayer of RPE cells was scratched using a 1000-μl pipette tip and cultured in the presence or absence of TNF-α/TGF-β_2_ and AICAR for 48 h. Representative photos are shown. Arrowheads indicate piled up cellular aggregates. Scale bars = 100 μm.
(PDF)

## References

[1] Leaver PK. Proliferative vitreoretinopathy. Br J Ophthalmol. 1995;79(10):871–872. 7488570 10.1136/bjo.79.10.871 PMC505283

[2] Newsome DA, Rodrigues MM, Machemer R. Human massive periretinal proliferation. In vitro characteristics of cellular components. Arch Ophthalmol. 1981;99(5):873–880. 7016092 10.1001/archopht.1981.03930010873017

[3] Garweg JG, Tappeiner C, Halberstadt M. Pathophysiology of proliferative vitreoretinopathy in retinal detachment. Surv Ophthalmol. 2013;58(4):321–329. doi: 10.1016/j.survophthal.2012.12.004 23642514 10.1016/j.survophthal.2012.12.004

[4] Lamouille S, Xu J, Derynck R. Molecular mechanisms of epithelial-mesenchymal transition. Nat Rev Mol Cell Biol. 2014;15(3):178–196. doi: 10.1038/nrm3758 24556840 10.1038/nrm3758 PMC4240281

[5] Lee JM, Dedhar S, Kalluri R, Thompson EW. The epithelial-mesenchymal transition: new insights in signaling, development, and disease. J Cell Biol. 2006;172(7):973–981. doi: 10.1083/jcb.200601018 16567498 10.1083/jcb.200601018 PMC2063755

[6] Nistico P, Bissell MJ, Radisky DC. Epithelial-mesenchymal transition: General principles and pathological relevance with special emphasis on the role of matrix metalloproteinases. Cold Spring Harb Perspect Biol. 2012;4(2):a011908–a011908. doi: 10.1101/cshperspect.a011908 22300978 10.1101/cshperspect.a011908 PMC3281569

[7] Casaroli-Marano RP, Pagan R, Vilaró S. Epithelial-mesenchymal transition in proliferative vitreoretinopathy: intermediate filament protein expression in retinal pigment epithelial cells.Invest Ophthalmol Vis Sci. 1999;40(9):2062–2072. 10440262

[8] Hiscott P, Sheridan C, Magee RM, Grierson I. Matrix and the retinal pigment epithelium in proliferative retinal disease. Prog Retin Eye Res. 1999;18(2):167–190. 9932282 10.1016/s1350-9462(98)00024-x

[9] Saika S, Yamanaka O, Flanders KC, Okada Y, Miyamoto T, Sumioka T, et al Epithelial-mesenchymal transition as a therapeutic target for prevention of ocular tissue fibrosis. Endocr Metab Immune Disord Drug Targets. 2008;8(1):69–76. 18393925 10.2174/187153008783928343

[10] Yang S, Li H, Li M, Wang F. Mechanisms of epithelial-mesenchymal transition in proliferative vitreoretinopathy. Discov Med. 2015;20(110):207–217. 26562474

[11] Hardie DG, Hawley SA. AMP-activated protein kinase: the energy charge hypothesis revisited. Bioessays. 2001;23(12):1112–1119. doi: 10.1002/bies.10009 11746230 10.1002/bies.10009

[12] Hawley SA, Davison M, Woods A, Davies SP, Beri RK, Carling D, et al Characterization of the AMP-activated protein kinase kinase from rat liver and identification of threonine 172 as the major site at which it phosphorylates AMP-activated protein kinase. J Biol Chem. 1996:271(44):27879–27887. 8910387 10.1074/jbc.271.44.27879

[13] Hardie DG. New roles for the LKB1—>AMPK pathway. Curr Opin Cell Biol. 2005;17(2):167–173. doi: 10.1016/j.ceb.2005.01.006 15780593 10.1016/j.ceb.2005.01.006

[14] Lee JH, Kim JH, Kim JS, Chang JW, Kim SB, Park JS, et al AMP-activated protein kinase inhibits TGF-β -, angiotensin II-, aldosterone-, high glucose-, and albumin-induced epithelial-mesenchymal transition. Am J Physiol Renal Physiol. 2013;304(6):F686–F697. doi: 10.1152/ajprenal.00148.2012 23324179 10.1152/ajprenal.00148.2012

[15] Qiu S, Xiao Z, Piao C, Zhang J, Dong Y, Cui W, et al AMPKα2 reduces renal epithelial transdifferentiation and inflammation after injury through interaction with CK2β. J Pathol. 2015;237(3):330–342. doi: 10.1002/path.4579 26108355 10.1002/path.4579

[16] Qu C, Zhang W, Zheng G, Zhang Z, Yin J, He Z. Metformin reverses multidrug resistance and epithelial-mesenchymal transition (EMT) via activating AMP-activated protein kinase (AMPK) in human breast cancer cells. Mol Cell Biochem. 2014;386(1–2):63–71. doi: 10.1007/s11010-013-1845-x 24096736 10.1007/s11010-013-1845-x

[17] Zhao Z, Cheng X, Wang Y, Han R, Li L, Xiang T, et al Metformin inhibits the IL-6-induced epithelial-mesenchymal transition and lung adenocarcinoma growth and metastasis. PLoS ONE. 2014;9(4):e95884 doi: 10.1371/journal.pone.0095884 24789104 10.1371/journal.pone.0095884 PMC4005743

[18] Lin H, Li N, He H, Ying Y, Sunkara S, Luo L, et al AMPK inhibits the stimulatory effects of TGF-β on Smad2/3 activity, cell migration, and epithelial-to-mesenchymal transition. Mol Pharmacol. 2015;88(6):1062–1071. doi: 10.1124/mol.115.099549 26424816 10.1124/mol.115.099549 PMC4658597

[19] Takahashi E, Nagano O, Ishimoto T, Yae T, Suzuki Y, Shinoda T, et al Tumor necrosis factor-α regulates transforming growth factor-β-dependent epithelial-mesenchymal transition by promoting hyaluronan-CD44-moesin interaction. J Biol Chem. 2010;285(6):4060–4073. doi: 10.1074/jbc.M109.056523 19965872 10.1074/jbc.M109.056523 PMC2823547

[20] Morizane Y, Thanos A, Takeuchi K, Murakami Y, Kayama M, Trichonas G, et al AMP-activated protein kinase suppresses matrix metalloproteinase-9 expression in mouse embryonic fibroblasts. J Biol Chem. 2011;286(18):16030–16038. doi: 10.1074/jbc.M110.199398 21402702 10.1074/jbc.M110.199398 PMC3091212

[21] Moysidis SN, Thanos A, Vavvas DG. Mechanisms of inflammation in proliferative vitreoretinopathy: from bench to bedside. Mediators Inflamm. 2012;2012(5):1–11. 10.1155/2012/815937 PMC3463807 23049173

[22] Saika S, Yamanaka O, Ikeda K, Kim-Mitsuyama S, Flanders KC, Yoo J, et al Inhibition of p38MAP kinase suppresses fibrotic reaction of retinal pigment epithelial cells. Lab Invest. 2005;85(7):838–850. doi: 10.1038/labinvest.3700294 15924151 10.1038/labinvest.3700294

[23] Xu K-P, Yu F-SX. Cross talk between c-Met and epidermal growth factor receptor during retinal pigment epithelial wound healing. Invest Ophthalmol Vis Sci. 2007;48(5):2242 doi: 10.1167/iovs.06-0560 17460286 10.1167/iovs.06-0560 PMC2215058

[24] He S, Kumar SR, Zhou P, Krasnoperov V, Ryan SJ, Gill PS, et al Soluble EphB4 inhibition of PDGF-induced RPE migration in vitro. Invest Ophthalmol Vis Sci. 2010;51(1):543–552. doi: 10.1167/iovs.09-3475 19696168 10.1167/iovs.09-3475 PMC2828363

[25] Chen YJ, Tsai RK, Wu WC, He MS, Kao Y-H, Wu WS. Enhanced PKCδ and ERK signaling mediate cell migration of retinal pigment epithelial cells synergistically induced by HGF and EGF. Yang C-M, ed. PLoS ONE. 2012;7(9):e44937 doi: 10.1371/journal.pone.0044937 23028692 10.1371/journal.pone.0044937 PMC3447816

[26] Chan C-M, Chang H-H, Wang V-C, Huang C-L, Hung C-F. Inhibitory effects of resveratrol on PDGF-BB-induced retinal pigment epithelial cell migration via PDGFRβ, PI3K/Akt and MAPK pathways. Yang C-M, ed. PLoS ONE. 2013;8(2):e56819 doi: 10.1371/journal.pone.0056819 23457620 10.1371/journal.pone.0056819 PMC3572951

[27] Xiao W, Chen X, Liu X, Luo L, Ye S, Liu Y. Trichostatin A, a histone deacetylase inhibitor, suppresses proliferation and epithelial-mesenchymal transition in retinal pigment epithelium cells. J Cell Mol Med. 2014;18(4):646–655. doi: 10.1111/jcmm.12212 24456602 10.1111/jcmm.12212 PMC4000116

[28] Saika S. TGFβ pathobiology in the eye. Lab Invest. 2005;86(2):106–115. 10.1038/labinvest.3700375 16341020

[29] Kita T, Hata Y, Arita R, Kawahara S, Miura M, Nakao S, et al Role of TGF-beta in proliferative vitreoretinal diseases and ROCK as a therapeutic target. Proc Natl Acad Sci USA. 2008;105(45):17504–17509. doi: 10.1073/pnas.0804054105 18952846 10.1073/pnas.0804054105 PMC2582249

[30] Laplante M, Sabatini DM. mTOR signaling at a glance. J Cell Sci. 2009;122(Pt 20):3589–3594. doi: 10.1242/jcs.051011 19812304 10.1242/jcs.051011 PMC2758797

[31] Qin S, Ni M, De Vries GW. Implication of S-adenosylhomocysteine hydrolase in inhibition of TNF-alpha- and IL-1beta-induced expression of inflammatory mediators by AICAR in RPE cells. Invest Ophthalmol Vis Sci. 2008;49(3):1274–1281. doi: 10.1167/iovs.07-1109 18326758 10.1167/iovs.07-1109

[32] Kuo C-L, Ho F-M, Chang MY, Prakash E, Lin W-W. Inhibition of lipopolysaccharide-induced inducible nitric oxide synthase and cyclooxygenase-2 gene expression by 5-aminoimidazole-4-carboxamide riboside is independent of AMP-activated protein kinase. J Cell Biochem. 2008;103(3):931–940. doi: 10.1002/jcb.21466 17615555 10.1002/jcb.21466

[33] Jhun BS, Jin Q, Oh YT, Kim SS, Kong Y, Cho YH, et al 5-Aminoimidazole-4-carboxamide riboside suppresses lipopolysaccharide-induced TNF-α production through inhibition of phosphatidylinositol 3-kinase/Akt activation in RAW 264.7 murine macrophages. Biochem Biophys Res Commun. 2004;318(2):372–380. doi: 10.1016/j.bbrc.2004.04.035 15120611 10.1016/j.bbrc.2004.04.035

[34] Hunt RC, Fox A, Pakalnis V, Sigel MM, Kosnosky W, Choudhury P, et al Cytokines cause cultured retinal pigment epithelial cells to secrete metalloproteinases and to contract collagen gels. Invest Ophthalmol Vis Sci. 1993;34(11):3179–3186. 8407227

[35] Eichler W, Friedrichs U, Thies A, Tratz C, Wiedemann P. Modulation of matrix metalloproteinase and TIMP-1 expression by cytokines in human RPE cells. Invest Ophthalmol Vis Sci. 2002;43(8):2767–2773. 12147614

[36] Kon CH, Occleston NL, Charteris D, Daniels J, Aylward GW, Khaw PT. A prospective study of matrix metalloproteinases in proliferative vitreoretinopathy. Invest Ophthalmol Vis Sci. 1998;39(8):1524–1529. 9660504

[37] Liou GI, Pakalnis VA, Matragoon S, Samuel S, Behzadian MA, Baker J, et al HGF regulation of RPE proliferation in an IL-1β/retinal hole-induced rabbit model of PVR. Mol Vis. 2002;8:494–501. 12500176

[38] Symeonidis C, Diza E, Papakonstantinou E, Souliou E, Karakiulakis G, Dimitrakos SA. Expression of matrix metalloproteinases in the subretinal fluid correlates with the extent of rhegmatogenous retinal detachment. Graefes Arch Clin Exp Ophthalmol. 2007;245(4):560–568. doi: 10.1007/s00417-006-0386-3 16941143 10.1007/s00417-006-0386-3

[39] Kauffmann DJ, van Meurs JC, Mertens DA, Peperkamp E, Master C, Gerritsen ME. Cytokines in vitreous humor: interleukin-6 is elevated in proliferative vitreoretinopathy. Invest Ophthalmol Vis Sci. 1994;35(3):900–906. 8125753

[40] Pennock S, Kim D, Mukai S, Kuhnle M, Chun DW, Matsubara J, et al Ranibizumab is a potential prophylaxis for proliferative vitreoretinopathy, a nonangiogenic blinding disease. Am J Pathol. 2013;182(5):1659–1670. doi: 10.1016/j.ajpath.2013.01.052 23582767 10.1016/j.ajpath.2013.01.052 PMC3644731

[41] Wang Q, Qi J, Hu R, Chen Y, Kijlstra A, Yang P. Effect of berberine on proinflammatory cytokine production by ARPE-19 Cells following stimulation with tumor necrosis factor-α. Invest Ophthalmol Vis Sci. 2012;53(4):2395–2402. doi: 10.1167/iovs.11-8982 22427564 10.1167/iovs.11-8982

[42] Kim J, Yoon MY, Choi SL, Kang I, Kim SS, Kim YS, et al Effects of stimulation of AMP-activated protein kinase on insulin-like growth factor 1- and epidermal growth factor-dependent extracellular signal-regulated kinase pathway. J Biol Chem. 2001;276(22):19102–19110. doi: 10.1074/jbc.M011579200 11262401 10.1074/jbc.M011579200

[43] Giri S, Nath N, Smith B, Viollet B, Singh AK, Singh I. 5-aminoimidazole-4-carboxamide-1-beta-4-ribofuranoside inhibits proinflammatory response in glial cells: a possible role of AMP-activated protein kinase. J Neurosci. 2004;24(2):479–487. doi: 10.1523/JNEUROSCI.4288-03.2004 14724246 10.1523/JNEUROSCI.4288-03.2004 PMC6729991

[44] Shibata T, Takaguri A, Ichihara K, Satoh K. Inhibition of the TNF-α-induced serine phosphorylation of IRS-1 at 636/639 by AICAR. J Pharmacol Sci. 2013;122(2):93–102. 23698110 10.1254/jphs.12270fp

