# Supplementary figures and images for: Suppressive effect of AMP-activated protein kinase on the epithelial-mesenchymal transition in retinal pigment epithelial cells
Source: PLoS One. 2017 Jul 18;12(7):e0181481. doi: 10.1371/journal.pone.0181481 (PMC5515442; doi:10.1371/journal.pone.0181481)

# S1 Fig

Control

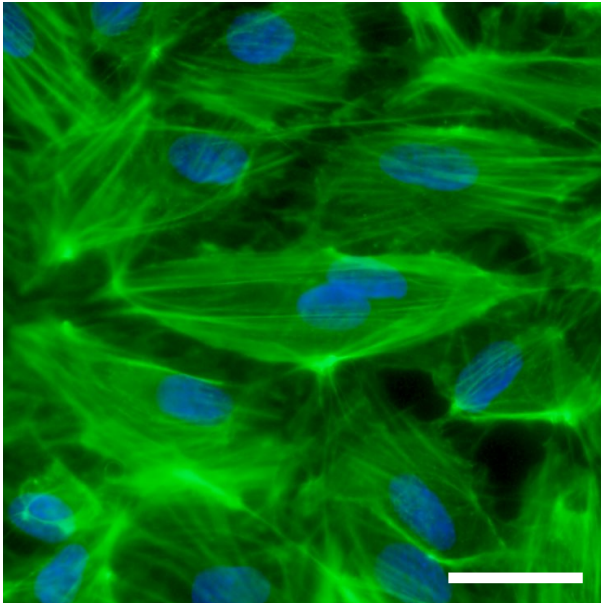

TNF- $\alpha$  + TGF- $\beta_2$

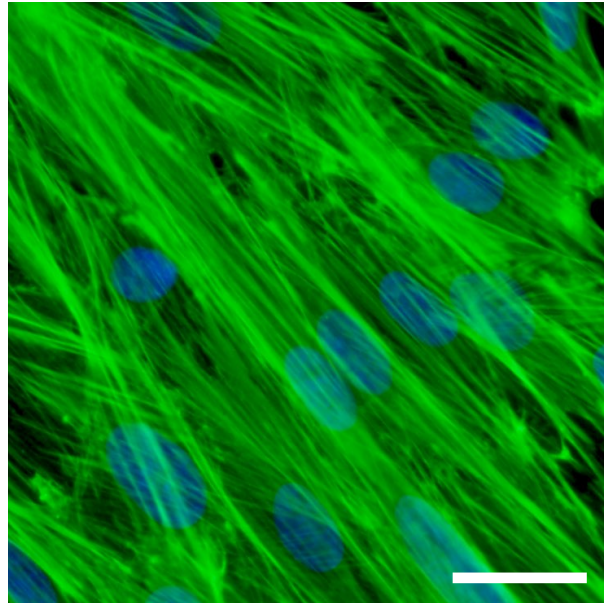

Supplement: S1 Fig — ARPE-19 cells were cultured with or without TNF-α/TGF-β2 for 48 h and stained with Acti-stain™ 488 phalloidin (Cytoskeleton, Inc.). Nuclei were counterstained with DAPI. Representative photos are shown. Scale bars = 20 μm. (PDF) [file pone.0181481.s001.pdf]

S2 Fig

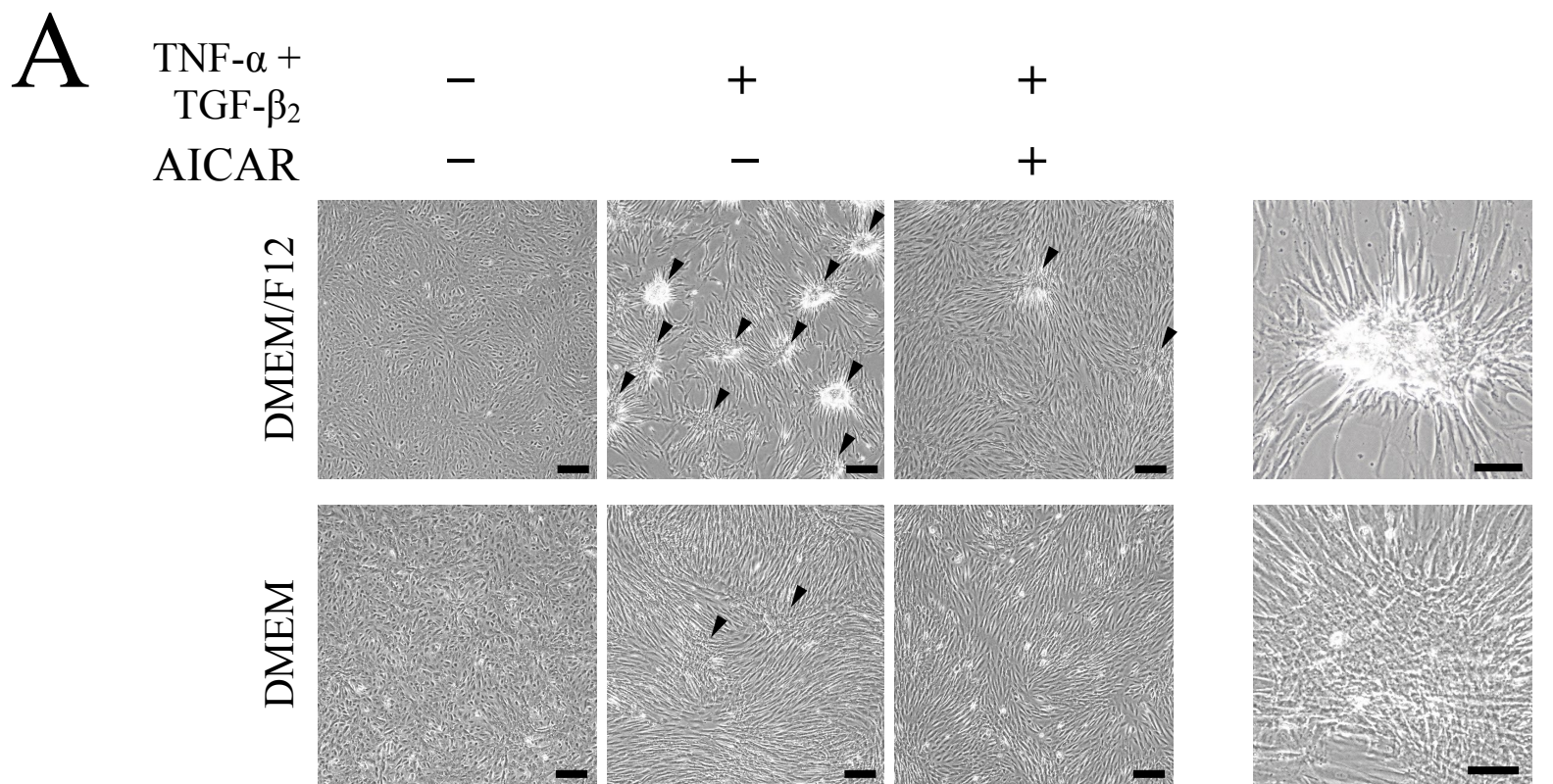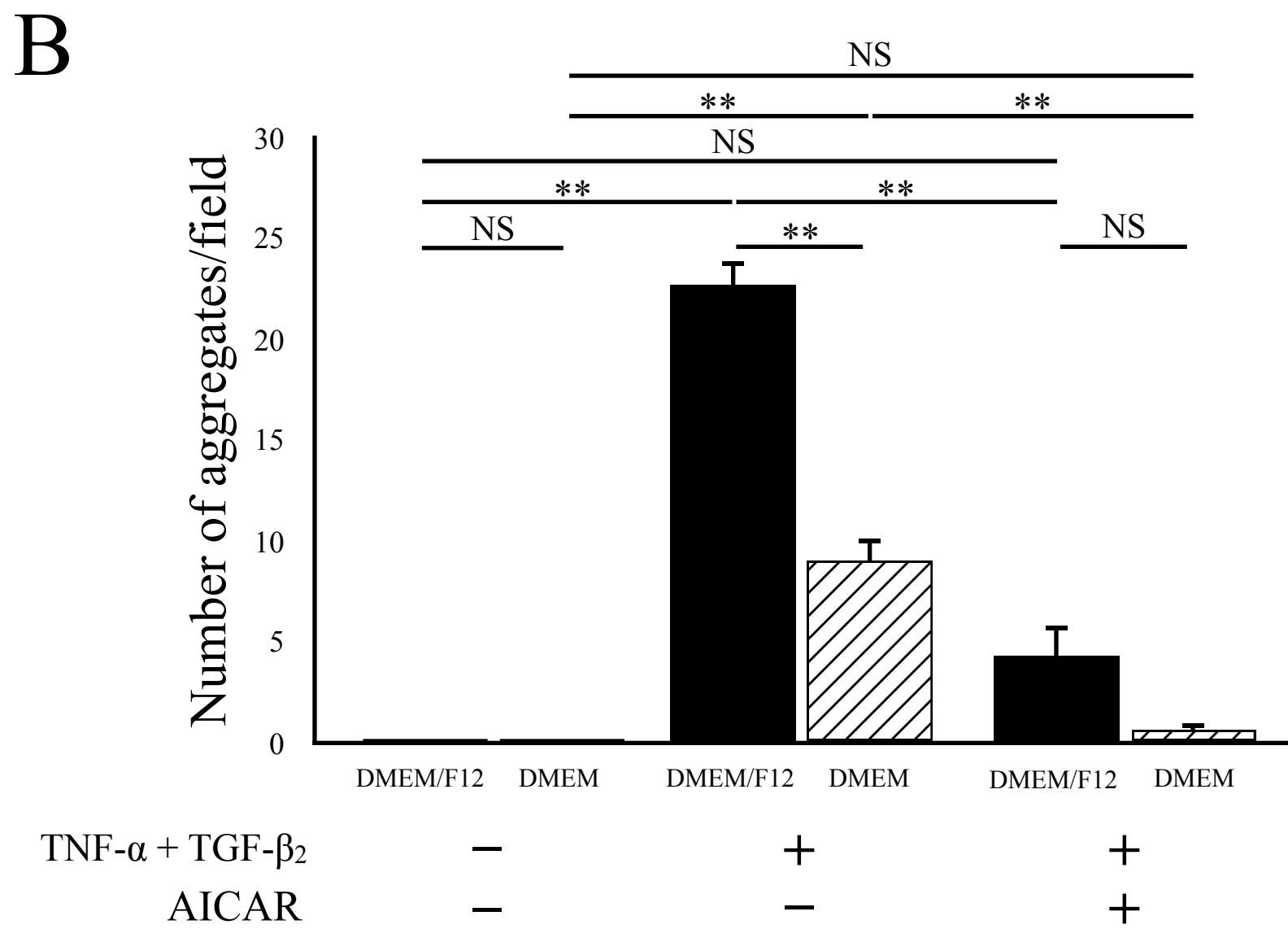

Supplement: S2 Fig — (A) ARPE-19 cells were cultured in the absence or presence of TNF-α/TGF-β2 and AICAR for 48 h in DMEM/F-12 or DMEM. Left, Representative images. Arrowheads indicate piled up cellular aggregates. Right, Magnified images of the aggregate. Scale bars = 200 μm (left images) and 50 μm (right magnified images). (B) Comparison of the number of TNF-α/TGF-β2-induced cellular aggregates with or without F-12. The number of aggregates per microscopic field was counted. **, p < 0.01. Error bars, S.E. (C) Comparison of the suppressive effect of AICAR on the TNF-α/TGF-β2-induced cellular aggregate formation with or without F-12. The number of aggregates per microscopic field was counted and analyzed. **, p < 0.01; NS, not significant. Error bars, S.E. (PDF) [file pone.0181481.s002.pdf]

# S3 Fig

A

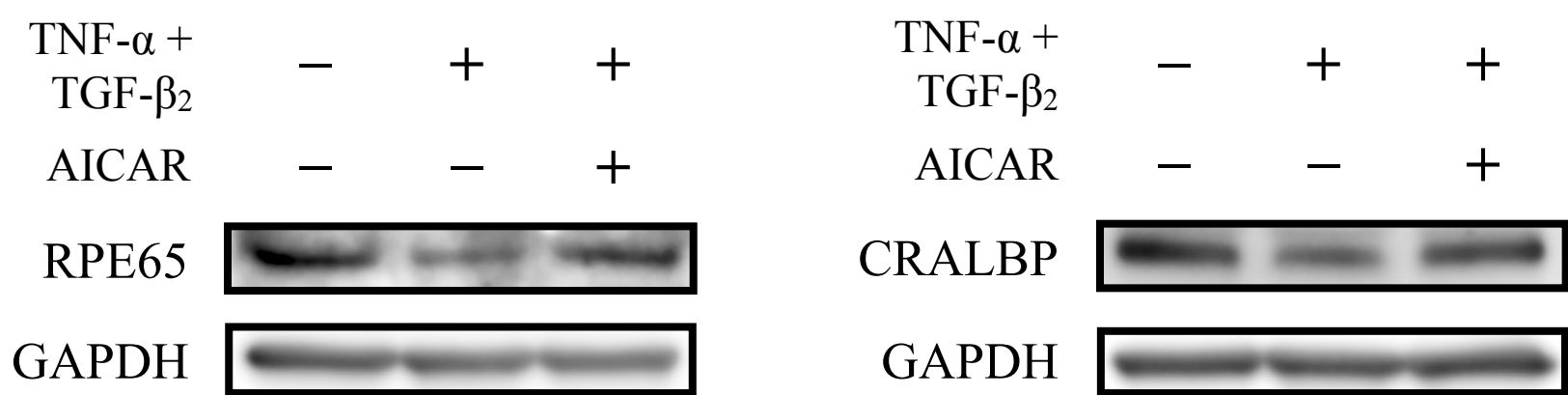

B

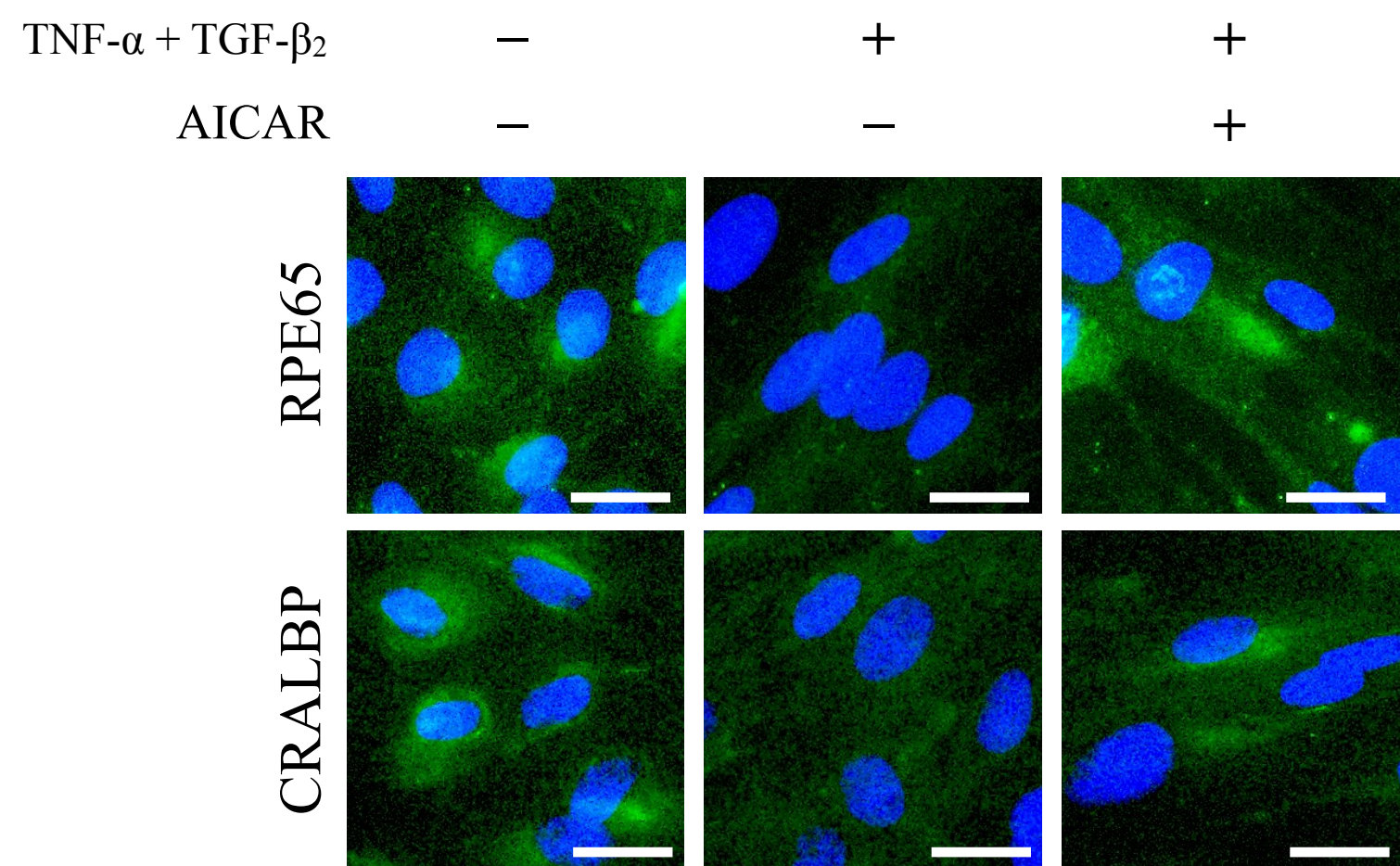

Supplement: S3 Fig — (A) Levels of RPE65 and CRALBP were determined by western blot analysis. GAPDH was used as a loading control. (B) ARPE-19 cells cultured with or without TNF-α/TGF-β2 and AICAR for 48 h were fixed and stained using antibodies against RPE65 and CRALBP (Abcam). Nuclei were counterstained with DAPI. Representative photos are shown. Scale bars = 20 μm. (PDF) [file pone.0181481.s003.pdf]

# S4 Fig

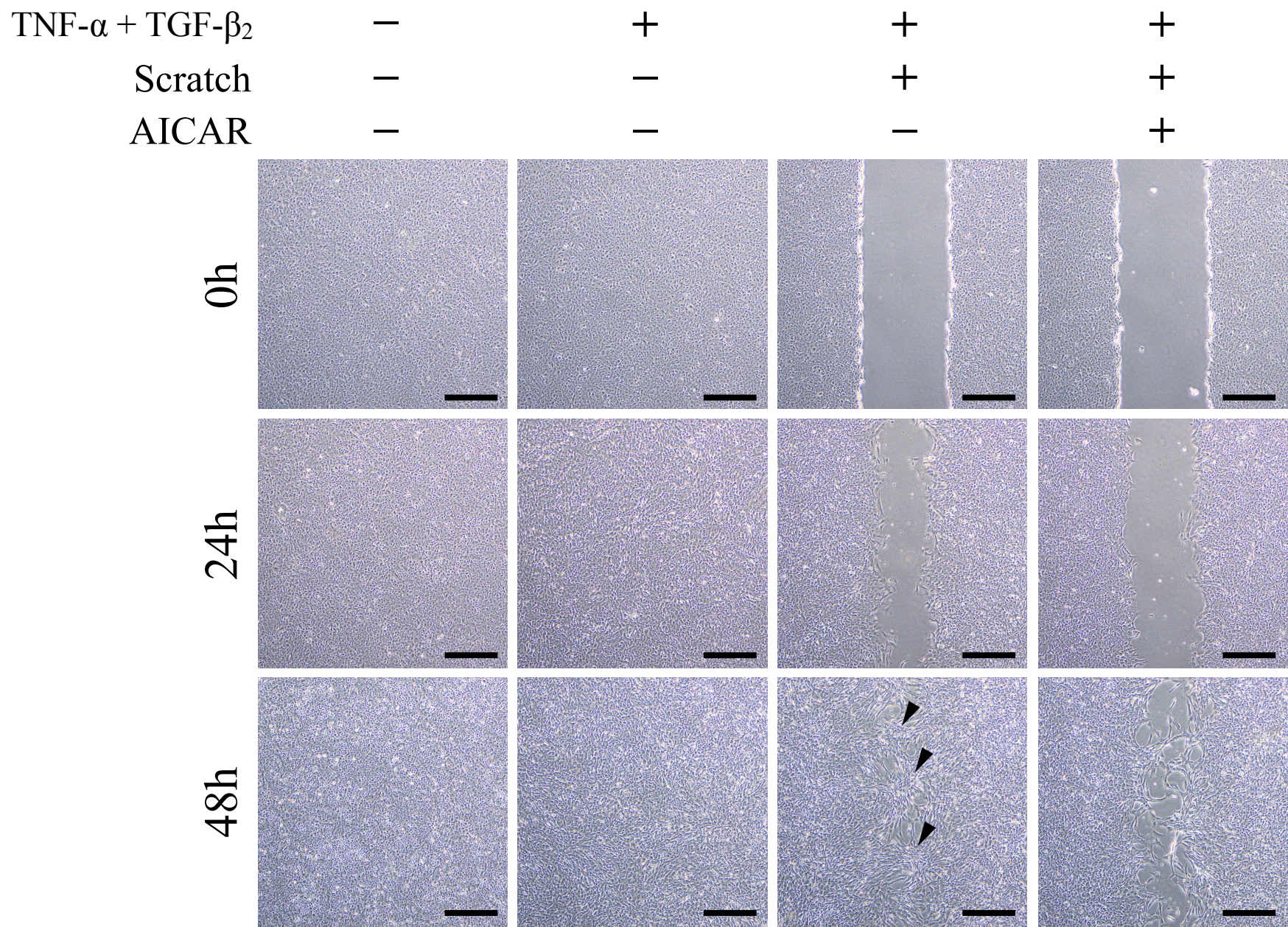

Supplement: S4 Fig — Primary RPE cells were cultured to confluence. The monolayer of RPE cells was scratched using a 1000-μl pipette tip and cultured in the presence or absence of TNF-α/TGF-β2 and AICAR for 48 h. Representative photos are shown. Arrowheads indicate piled up cellular aggregates. Scale bars = 100 μm. (PDF) [file pone.0181481.s004.pdf]
